# Variation of Vegetation Ecological Water Consumption and Its Response to Vegetation Coverage Changes in the Rocky Desertification Areas in South China

**DOI:** 10.1371/journal.pone.0163566

**Published:** 2016-10-31

**Authors:** Long Wan, Jing Tong, Jinxing Zhou, Hongyan Guo, Ming Cui, Yuguo Liu, Like Ning, Fukai Tang

**Affiliations:** 1 Jianshui Karst Ecosystem of the National Field Research Station, Key Laboratory of State Forestry Administration on Soil and Water Conservation, Beijing Forestry University, Beijing, China; 2 The College of Forestry, Beijing Forestry University, Beijing, China; 3 Institute of Desertification Studies, Chinese Academy of Forestry, Beijing, China; 4 Key Laboratory of Water Cycle & Related Land Surface Processes, Institute of Geographic Sciences and Natural Resources Research, Chinese Academy of Sciences, Beijing, China; 5 Graduate University of Chinese Academy of Sciences, Beijing, China; Beijing Normal University, CHINA

## Abstract

Over the past several decades, rocky desertification has led to severe ecological problems in karst areas in South China. After a rocky desertification treatment project was completed, the vegetation coverage changed greatly and, consequently, increased the ecology water consumption (approximately equal to the actual evapotranspiration) of the regional vegetation. Thus, it intensified the regional water stresses. This study explored the changes in the actual evapotranspiration (ETa) response to the vegetation coverage changes in the rocky desertification areas in South China based on the precipitation (P), potential evapotranspiration (ETp) and NDVI (the normalized difference vegetation index) datasets. The revised Bagrov model was used to simulate the actual evapotranspiration changes with the supposed increasing NDVI. The results indicated that the average NDVI value was lower when the rocky desertification was more severe. The ETa, evapotranspiration efficiency (ETa/ETp) and potential humidity (P/ETp) generally increased with the increasing NDVI. The sensitivity of the ETa response to vegetation coverage changes varied due to different precipitation conditions and different rocky desertification severities. The ETa was more sensitive under drought conditions. When a drought occurred, the ETa exhibited an average increase of 40~60 mm with the NDVI increasing of 0.1 in the rocky desertification areas. Among the 5 different severity categories of rocky desertification, the ETa values’ responses to NDVI changes were less sensitive in the severe rocky desertification areas but more sensitive in the extremely and potential rocky desertification areas. For example, with the NDVI increasing of 0.025, 0.05, 0.075, and 0.1, the corresponding ETa changes increased by an average of 2.64 mm, 10.62 mm, 19.19 mm, and 27.58 mm, respectively, in severe rocky desertification areas but by 4.94 mm, 14.99 mm, 26.80, and 37.13 mm, respectively, in extremely severe rocky desertification areas. Understanding the vegetation ecological water consumption response to the vegetation coverage changes is essential for the vegetation restoration and water stresses mitigation in rocky desertification areas.

## Introduction

In the karst areas of South China, intensive vegetation degradation and severe rocky desertification have threatened the local ecological security [1]. Vegetation restoration is crucial for improving the regional eco-environmental quality [2]. Thus, large areas of artificial vegetation were established in the rocky desertification areas. In particular, after 2008, the rocky desertification treatment project was conducted by the government and greatly improved the local ecological environment, and large changes in the vegetation coverage occurred in this region [3].

Water has been a key factor limiting the eco-restoration and construction of the vegetation [4, 5]. As the vegetation coverage increased, the vegetation ecological water consumption was likely to rise [6, 7]. However, because of the frequent drought occurrence, the thin soil, and the extensively exposed bedrock in rocky desertification areas, the storage capacity of water in the soil layers was low, which resulted in the deficiency of water for vegetation growth [8, 9]. The increasing water demand for the new artificial vegetation will be difficult to derive from the available surface and soil water [10, 11]. The increase in vegetation coverage intensified the water stresses in this region [12]. Understanding the vegetation ecological water consumption response to the vegetation coverage changes is essential for rational ecological construction and ecological sustainable development in rocky desertification areas.

Actual evapotranspiration (ETa) by plants represents the major proportion of vegetation ecology water consumption in a regional ecosystem [13]. It contains the sum of the soil and canopy evaporation and plants transpiration, and it accounts for more than 95% of the vegetation ecology water consumption. Therefore, we used the amount of actual evapotranspiration to represent the vegetation ecology water consumption. Many studies showed that the ETa was mainly determined by the precipitation, vegetation coverage and reference evapotranspiration [14]. The variations in precipitation and vegetation coverage can lead to different responses to ETa changes.

Previous studies have focused on the research of actual evapotranspiration variability. In the upper Yangtze River, research results indicated that the ETa presented a significant decreasing trend in plain regions and an increasing trend in the mountainous region. After the 1990s, the average ETa dramatically declined in the upper Yangtze River [15, 16]. In the moderate reaches of the Yangtze River basin, the downward trends of ETa were more significant [17, 18]. In a severe rock desertification province, Guizhou Province, the ETa also significantly decreased at the significance level of 5% [19]. A few studies found some relationship between the ETa variability and the vegetation changes in South China. These studies concluded that afforestation efforts contributed to the vegetation coverage increase in northwestern Yunnan Province. A multivariate linear regression analysis indicated that the factor of vegetation coverage variation explained 14.82% of the ETa variation in this region [20].

ETa can be estimated by various methods [21–24]. Combining satellite NDVI (the Normalized Difference Vegetation Index) could enhance the accuracy of ETa measurements [25]. NDVI is an important index to reflect the degree of vegetation coverage [26]. Many researchers have studied the relationship between NDVI and ETa to understand the response of the ETa to the vegetation coverage variation. The results suggested that NDVI was an important variable for indirectly monitoring ETa over large areas [27], and there existed a positive linear relationship between the two datasets [28]. Researches in China also concluded that the integrated NDVI can be used to effectively estimate annual ETa in the Yellow River Basin [29].

However, the severity of the rocky desertification varied in South China and, consequently, the response of the ETa to vegetation coverage changes were different. In previous studies, the relationship between the ETa variation and the NDVI changes were not clear in the different rocky desertification areas. Our study focused on the ETa response to NDVI changes in the different severity categories of the rocky desertification areas. In addition, we determined what the increase in ETa would be if the vegetation restoration continued to increase to an assumed amount. This study will benefit for water resources planning in different rocky desertification areas, and for ecological construction in this area, it is useful for the evaluation of ecological water requirements.

## Study Area

The study area is located in the rocky desertification area in South China, which is distributed into 7 provinces and 1 municipality (which was considered a province), encompassing Sichuan, Yunnan, Guizhou, Hubei, Hunan, and Guangdong provinces, Chongqing Municipality, and Guangxi Zhuang Autonomous Region. The rocky desertification area coverage is approximately 1.2 million km^2^, which accounts for approximately 26.5% of the karst areas and 11.2% of the total areas of the 8 provinces. This area is one of the most ecologically fragile zones in China [30]. Rocky desertification is seriously constraining the sustainability of local development. The degeneration of forests and the decrease of regional vegetation have caused severe ecology problems and have resulted in the extensive exposure of the carbonate bedrocks. The plants here are mostly xerophilous vines, thorn bushes, coarse grass and succulent shrubs [31], and the predominant soil types are limestone soil, yellow soil, red soil, and purple soil. The rocky desertification areas occur mainly in two major river basins, the Yangtze River and Pearl River, accounting for 58.0% and 35.5% of the total areas, respectively. The average annual precipitation in this region is approximately 1300 mm and is unevenly distributed. The highest precipitation is more than 2000 mm, and the lowest is less than 700 mm. The potential evapotranspiration in most parts of the region is more than 1000 mm.

**Fig 1:**
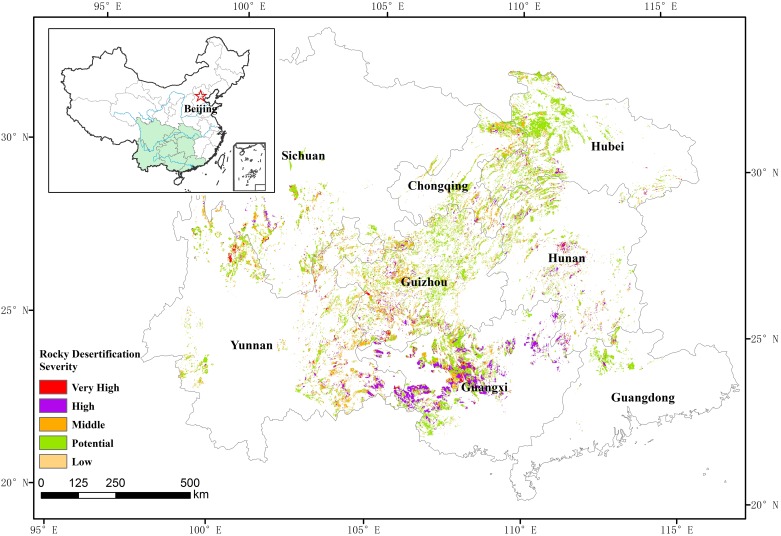
Spatial distribution of the different severity of the rocky desertification in the study area.

The severity of rocky desertification is commonly classified into 5 categories in South China: (1) potential rocky desertification; (2) light rocky desertification; (3) moderate rocky desertification; (4) severe rocky desertification; and (5) extremely severe rocky desertification (Fig 1). Many researchers have studied how to classify the severity of rocky desertification [32–35]. In this study, we choose these four commonly used indices to assess the severity of the rocky desertification, which were already been used in the monitoring of the rocky desertification in South China. The indices were exposed bedrock, vegetation coverage, vegetation type, and soil thickness. The rocky desertification classification was mainly divided according to the experts marking method (Table 1). The severity of rocky desertification assessment was based on the total mark of four indices. The method was shown as in Table 2.

**Table 1:** Marking standard of the indices for assessing rocky desertification severity.

Coverage rates of exposed bedrock(%)	30–39%	40–49%	50–59%	60–69%	≥70%
mark	20	26	32	38	44
Vegetation type	Trees	Shrubs	Grassland	Farmland	No vegetation
mark	5	8	12	16	20
Vegetation coverage rates	50–69%	30–49%	20–29%	10–19%	<10%
mark	5	8	14	20	26
Soil thickness	≥40cm	20–39cm	10–19cm	<10cm	
mark	1	3	6	10	

**Table 2:** Classification of the rocky desertification severity.

Severity of rocky desertification	Coverage rates of exposed bedrock	Vegetation coverage rates	Total mark of the four indices
potential	≥30%	Trees, shrubs: ≥50	
		Grasslands: ≥70%	
light			≤45
moderate		Trees, shrubs: <50	46~60
severe		Grasslands: <70%	61~75
extremely severe			>75

## Materials and Methods

### Materials

The annual precipitation data records from 2000–2013 at 186 gauging stations were derived, which was interpolated by the Kriging method. We used the NDVI for July 11^th^ from 2000 to 2013, when the vegetation was growing well, for the ETa analysis and modeling. A series of processes, such as atmospheric correction, radiometric correction, and geometric correction, were completed to ensure data quality.

The MOD16 datasets were estimated using Mu et al.’s improved ET algorithm. The datasets was good to reflect the ETa variation [36, 37]. For ET evaluation at eddy flux towers, the improved algorithm reduces mean absolute bias (MAE) of daily ETa to 0.33 mm day^−1^ driven by tower meteorological data [38]. The evapotranspiration product contained all of the transpiration by vegetation and evaporation from canopy and soil surfaces. The main datasets used in this study were showed in Table 3.

**Table 3:** Main datasets sources.

Data	Data Source	Resolution
Precipitation	from the China Meteorological Administration (http://data.cma.cn/)	1km*1km
NDVI	SPOT VEGETATION 10-day Synthesis Archive (SPOT VGT-S10) NDVI products from the Cold and Arid Regions Sciences Data Center (http://westdc.westgis.ac.cn/)	1km*1km
ETa, ETp	The MOD16 global actual evapotranspiration (ETa)/potential evapotranspiration (ETp) datasets (ftp://ftp.ntsg.umt.edu/pub/MODIS/NTSG_Products/MOD16/MOD16A2_MONTHLY.MERRA_GMAO_1kmALB/GEOTIFF_0.05degree/)	0.05°*0.05°

### Methods

The Bagrov model can be applied to calculate the actual evapotranspiration. Bagrov first attempted to derive the analytical equation for the mean annual water-energy balance [39]. He analyzed the data on water balances and derived the Bagrov equation, in which evapotranspiration depended on the precipitation and potential evapotranspiration. The equation considered both water limiting and energy limiting for actual evapotranspiration [40]. In the case of arid conditions, water availability dominates evapotranspiration, and in the opposite condition, energy availability is crucial [41]. Thus, actual evapotranspiration is strongly limited by either water or energy availability. The functional relationship between the evapotranspiration efficiency (ETa/ETp) and the potential humidity (P/ETp) is given by the Bagrov equation (Eq 1) [42]. The equation directly reflects the Ea changes’ responses to precipitation and ETp changes.

$$\frac{dETa}{dP}=1-{\left(\frac{ETa}{ETp}\right)}^{N}$$ (1)

$$\;\to \frac{dETa}{dETp}*\frac{dETp}{dP}=1-{\left(\frac{ETa}{ETa}\right)}^{N}$$ (2)

$$\to \;d(\frac{ETa}{ETp})=(1-{\left(\frac{ETa}{ETp}\right)}^{N})*d(\frac{P}{ETp})$$ (3)

where N is determined by the vegetation conditions. Eq 3 can be solved by the numerical method [43]. In our study, the value of N was different under different NDVI conditions. K. Miegel et al. has also illustrated the relationship between the evapotranspiration efficiency (ETa/ETp) and the potential humidity (P/ETp) for given different values of N.

However, the actual evapotranspiration was considerably influenced by the vegetation coverage. Thus, we considered the impacts of the vegetation coverage, added the variable NDVI to the equation and revised the Bagrov model to simulate the ETa values. The equation was revised as follows:

$$d(\frac{ETa}{ETp})=(1-{\left(\frac{ETa}{ETp}\right)}^{N})*d(\frac{PAW*NDVI*P}{ETp})$$ (4)

where *Paw* is a coefficient determined by the model calibration with observed MODIS data. In our study, it was determined by the different vegetation conditions and different severity categories of rocky desertification.

The Nash–Sutcliffe efficiency (NSE) index [44] is one of the most commonly employed indices to evaluate the performance of a model [45–47]. It is defined as

$$E=1-\frac{\sum_{t=1}^{T}{\left(Q_{0}^{t}-Q_{m}^{t}\right)}^{2}}{\sum_{t=1}^{T}{\left(Q_{0}^{t}-\bar{Q_{0}}\right)}^{2}}$$ (5)

where *Q*_*0*_ is the observed value and *Q*_*m*_ is the modeled value. $Q_{0}^{t}$ is the observed value at time t. $\bar{Q_{0}}$ is the average value of the Q_0_. The NSE index is a dimensionless index that measures the model's efficiency at producing an index ranging from−∞to 1. The closer the model efficiency is to 1, the more accuracy the model provides. An efficiency of 0 indicates that the model predictions are as accurate as the mean of the observed data, whereas efficiency less than 0 occur when the observed mean is a better predictor than the model [48].

## Results

### Distribution and variation of precipitation, NDVI and vegetation ecological water consumption in rocky desertification areas in South China

**Fig 2:**
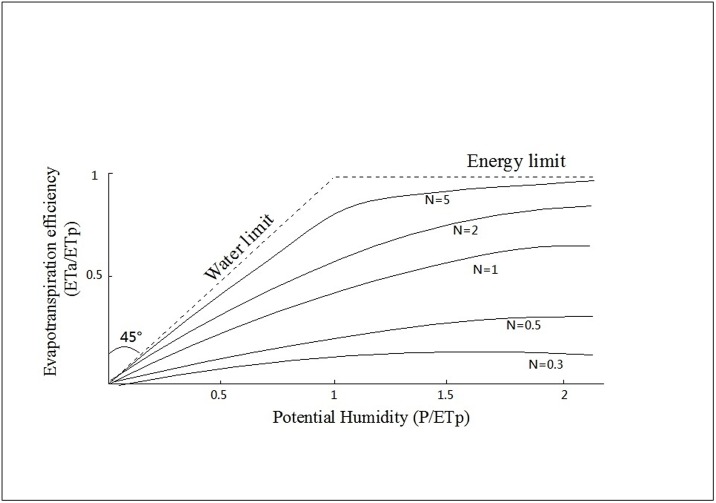
The spatial distribution of the mean annual precipitation in the rocky desertification areas in South China from 2000~2013.

The mean annual precipitation in rocky desertification areas in South China was approximately 1323.3 mm and ranged from 654.7 mm to 2272.2 mm (Fig 2). The distribution of precipitation was uneven in the rocky desertification areas. The degree of vegetation degradation also varied due to the uneven climate and topography conditions and to the different intensities of human activities. According to the Spot NDVI products, the results indicated that the NDVI varied between different rocky desertification regions. As a result, in July, the average NDVI value was 0.570 in this area. The lowest NDVI values were located in the in the arid valley areas in southern Sichuan Province and eastern Yunnan Province. The average annual ETa values in the rocky desertification areas ranged from 286 mm to 1072 mm, and the average annual ETp values ranged from 922 mm to 1084 mm. The spatial patterns of the ETa distribution and the amount of precipitation were similar in the rocky desertification areas.

**Fig 3:**
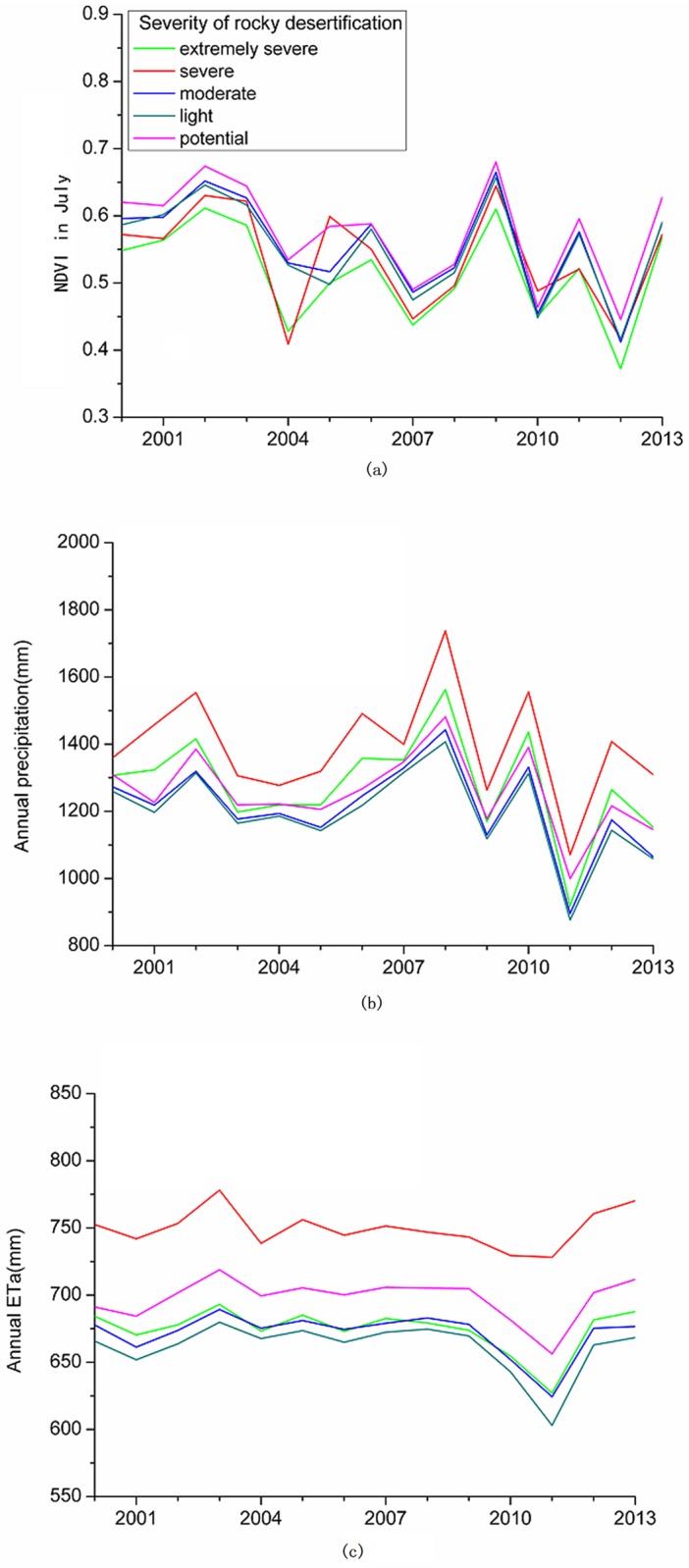
Annual variation of (a) NDVI in July, (b) actual evapotranspiration (ETa), and (c) average precipitation (P) in different severity categories of rocky desertification areas from 2000–2013.

The annual variations of precipitation, ETa, and NDVI in each rocky desertification area were similar. The average annual NDVI in July presented a decreasing trend from 2000 to 2013 in all of the rocky desertification areas, ranging from 0.37 to 0.68 (Fig 3a). No significant trends were observed for precipitation or ETa in the rocky desertification areas from 2000–2013 (Fig 3b and 3c). The average precipitation, NDVI and ETa in each rocky desertification area varied with the different severity categories of rocky desertification. With the increasing severity of rocky desertification, the NDVI value decreased. In the extremely severe and severe rocky desertification areas, the NDVI values were 0.5160 and 0.5381, respectively. However, in the other three areas, the NDVI value were more than 0.5500, with the highest NDVI value observed in the potential rocky desertification areas, which was 0.5780 (Table 4). The precipitation and ETa were lower in the moderate and light rocky desertification areas but higher in the severe rocky desertification areas.

**Table 4:** Mean values of the average precipitation, NDVI, and ETa in different severity categories of rocky desertification areas.

Degree of rocky desertification	Precipitation	NDVI	ETa
extremely severe	1278.4	0.5160	674.5
Severe	1393.4	0.5381	749.7
Moderate	1210.5	0.5581	671.5
Light	1193.8	0.5521	661.4
Potential	1256.6	0.5780	697.7

### Identification of the relationship among ETa, precipitation and NDVI variation in rocky desertification areas

**Fig 4:**
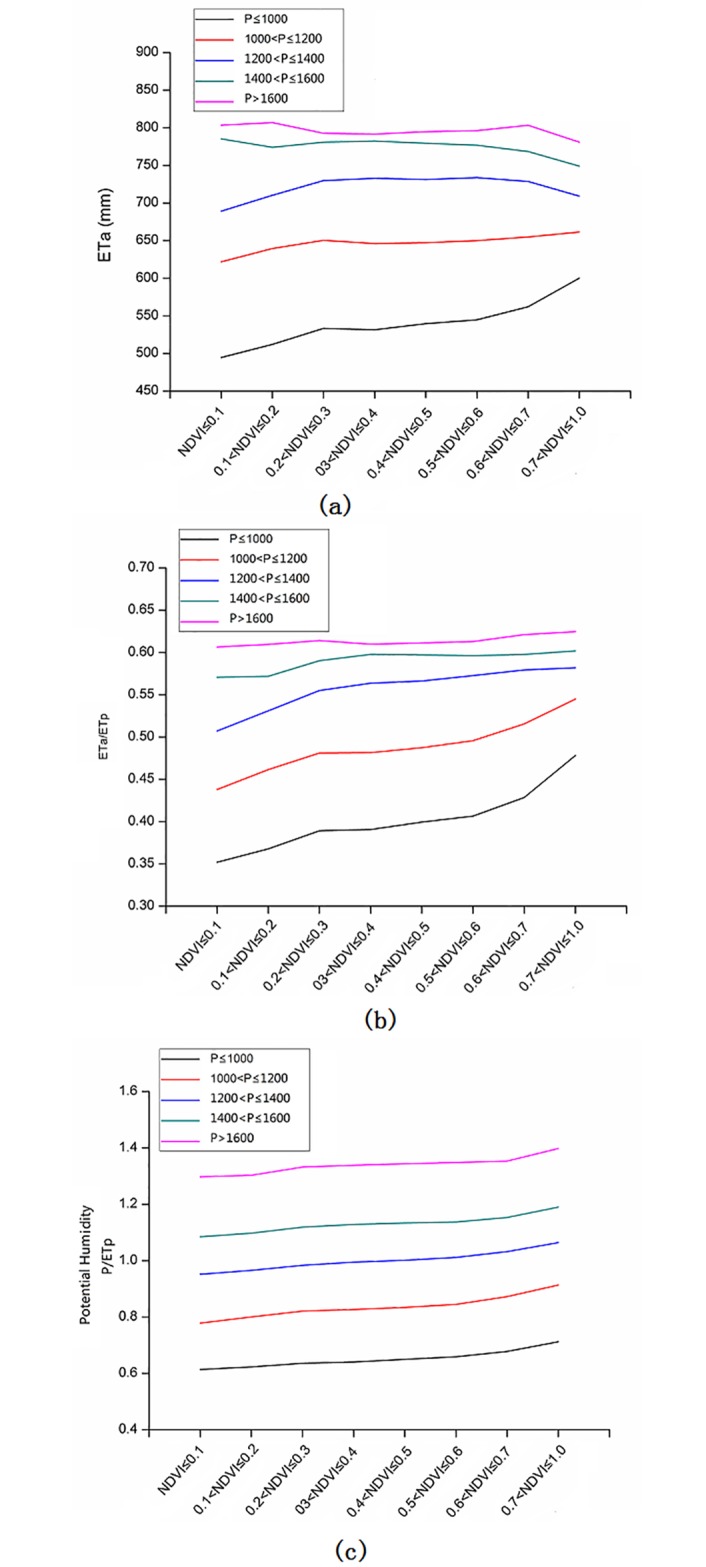
The (a) actual evapotranspiration (ETa), (b) evapotranspiration efficiency (ETa/ETp) and (c) potential humidity (P/ETp) responses to the NDVI and precipitation changes.

The vegetation ecological water consumption was greatly influenced by the precipitation, NDVI and ETp variation. It is very important to recognize the relationship among the ETa changes, precipitation, and NDVI changes under the vegetation restoration in rocky desertification areas. In this study, 8 classifications were used for the NDVI values, and 5 classifications were used for the precipitation amounts, as shown in Table 5. To investigate the relationship among these variables, the mean values of precipitation, ETp, ETa and NDVI were calculated for each precipitation classification and each NDVI classification. As a result, for the given classification of the P≤1000, 1000<P≤1200 or 1200<P≤1400, with the NDVI values increasing, the mean ETa values significantly increased (Fig 4a). However, for the given classifications of 1400<P≤1600 or 1600<P, the changes in the mean ETa values were not significantly different with the increasing NDVI. Furthermore, we explored the evapotranspiration efficiency (ETa/ETp) and the potential humidity (P/ETp) values responses to the NDVI changes. Obviously, with the NDVI increasing, the evapotranspiration efficiency and the potential humidity increased for all 5 precipitation classifications. This result indicated that both the evapotranspiration efficiency and the potential humidity had relatively positive responses to the vegetation coverage changes (Fig 4b and 4c). The increases in evapotranspiration efficiency and potential humidity were also more sensitive for the lower precipitation values.

**Table 5:** Classification of NDVI and precipitation amounts in our study.

Code of NDVI Classification	NDVI Classification	Code of Precipitation Classification	NDVI Classification
A	NDVI≤0.1	A	P≤1000
B	0.1<NDVI≤0.2	B	1000<P≤1200
C	0.2<NDVI≤0.3	C	1200<P≤1400
D	0.3<NDVI≤0.4	D	1400<P≤1600
E	0.4<NDVI≤0.5	E	P>1600
F	0.5<NDVI≤0.6		
G	0.6<NDVI≤0.7		
H	0.7<NDVI≤1		

**Fig 5:**
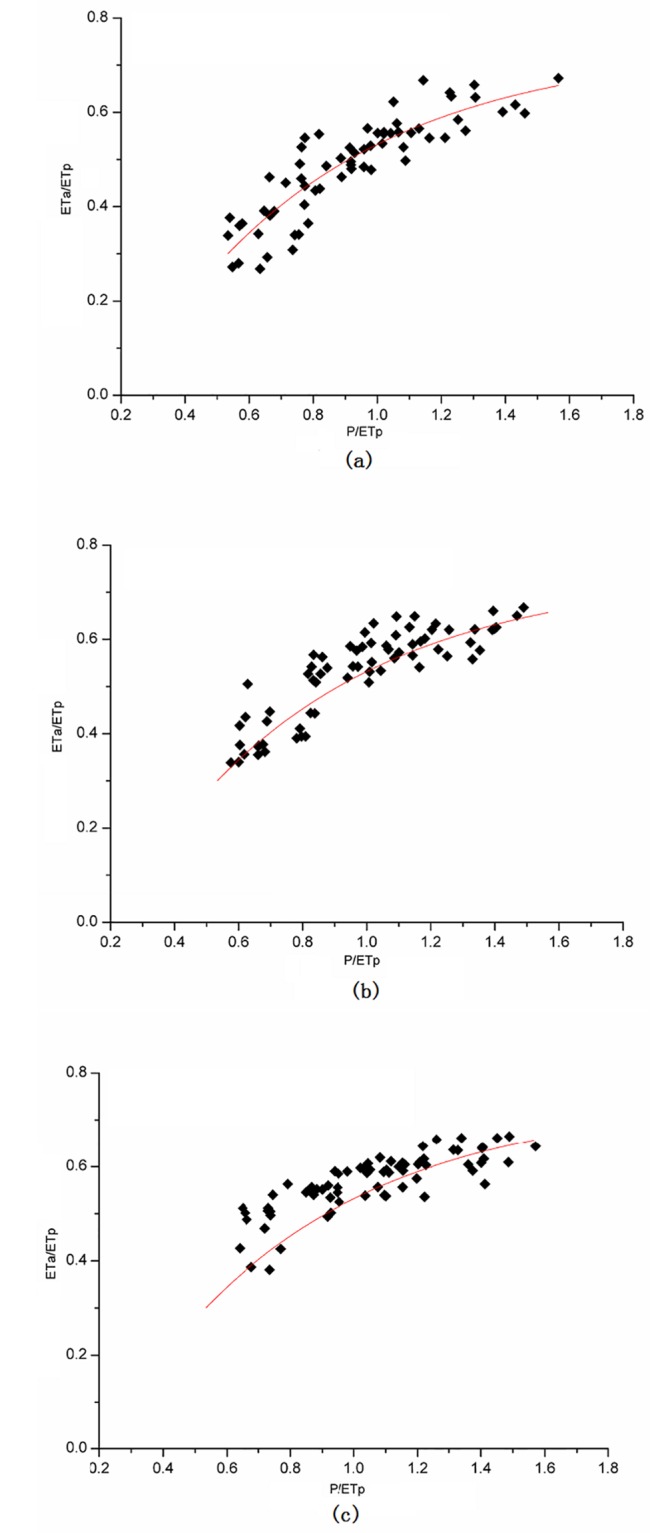
Relationship between evapotranspiration efficiency (ETa/ETp) and humidity (P/ETp) for different NDVI classifications: (a) NDVI≤0.1; (b) 0.3<NDVI≤0.4; and (c) 0.7<NDVI≤1.

We explored the relationship between the ETa/ETp and the P/ETp to ensure that the Bagrov model was suitable for simulating the ETa in the rocky desertification areas. The results indicated that the mean ETa/ETp and the mean P/ETp values for each given NDVI classification had a very similar relationship as the Bagrov model shown in the reference [41] (Fig 5a, 5b and 5c). Obviously, it was suitable for the Bagrov modeling of ETa. Additionally, we explored the ETa/ETp and the P/ETp values responses to the NDVI changes. We concluded that for all of the precipitation classifications, the ETa/ETp and the P/ETp values significantly increased with the increasing NDVI. Therefore, we revised the Bagrov model, obtained the parameter of NDVI, and used the revised Bagrov model to simulate the value of ETa.

### Vegetation ecological water consumption modeling and its response to vegetation coverage changes in different degrees of rocky desertification areas

The precipitation was still divided into 5 classifications, and the NDVI amount was still divided into 8 classifications, as in Table 3. In each severity category of the rocky desertification areas, the mean values of precipitation, ETp, ETa and NDVI were calculated for each precipitation classification and for each NDVI classification from 2000–2013. Then, in different NDVI classifications and different severity levels of the rocky desertification conditions, the mean ETa values were simulated using the Bagrov model. We used the MODIS estimated ETa values empirically to calibrate the parameters (PAW and N) and validate the results, combined with direct search method for optimization [49, 50].

**Fig 6:**
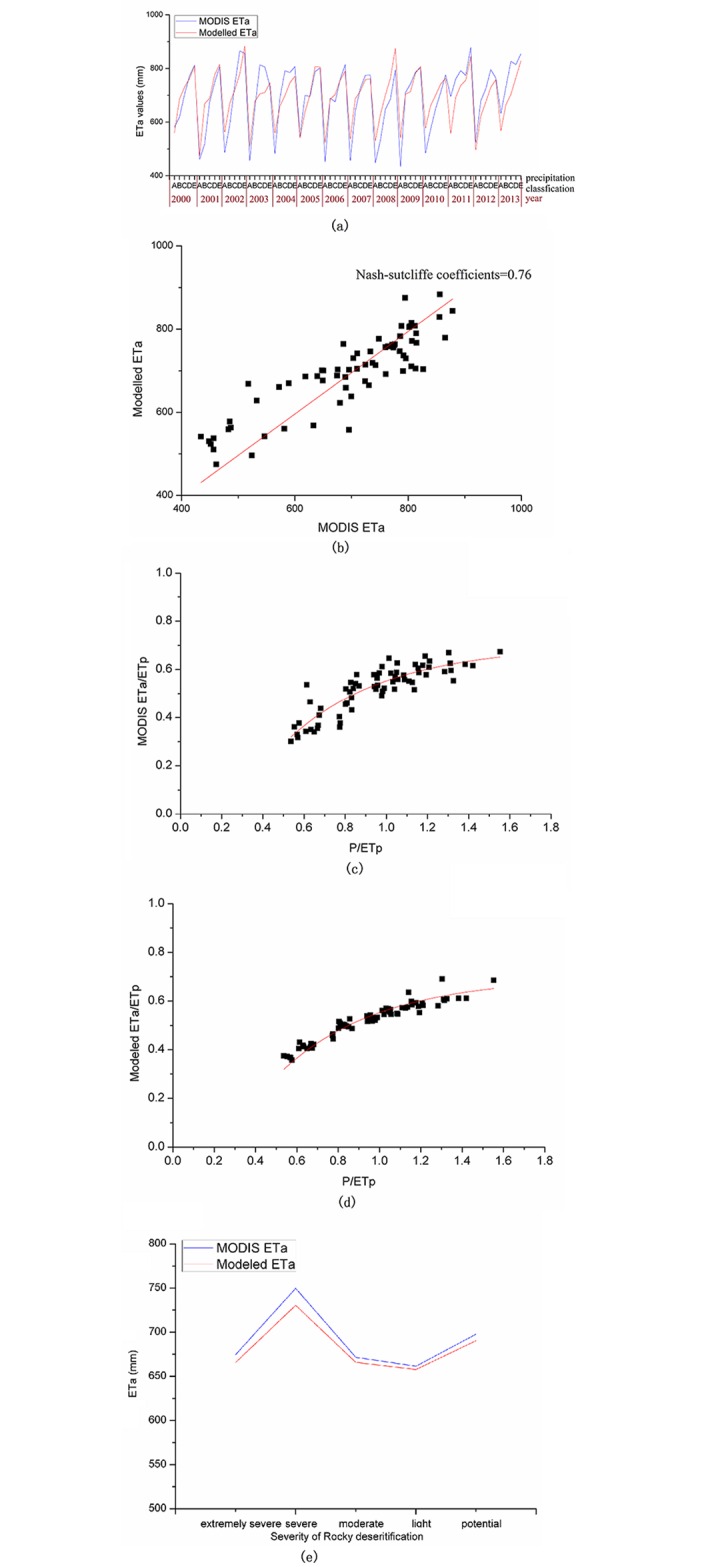
For the given NDVI classification of 0.3<NDVI≤0.4 in the moderate rocky desertification areas, (a) and (b), the comparison of ETa modeling results and the MODIS ETa values, respectively, and the comparison of the relationship between (c) (MODIS ETa)/ETp and (P/ETp), (d) (Modeled ETa)/ETp and the (P/ETp); and (e) the average annual ETa modeling results compared with the MODIS ETa for each rocky desertification area.

The Bagrov model parameters of PAW and N are shown in Table 6. The PAW values ranged from 0.92 to 8.40, and the N values ranged from 1.8 to 5. The results indicated that all of the Nash-Sutcliffe coefficients between the simulated ETa values and MODIS estimated ETa values were more than 0.70 for a given NDVI classification. For example, the ETa modeling results for the classification of 0.3<NDVI≤0.4 in the moderate rocky desertification area are shown in Fig 6a and 6b. The Bagrov model also correctly reflected the relationship between the evapotranspiration efficiency (ETa/ETp) and the potential humidity (P/ETp) (Fig 6c and 6d). The average annual ETa amount in the different rocky desertification areas from 2000–2013 was calculated. The corresponding error of the simulated average ETa in the potential, light, moderate, severe, and extremely rocky desertification areas were only -8.9 mm, -19.2 mm, -5.7 mm, -3.8 mm and -7.4 mm, respectively (Fig 6e). The revised Bagrov model was suitable to identify the ETa values’ changes in response to the precipitation and NDVI changes.

**Table 6:** Parameters (PAE and N) of the revised Bagrov modeling of ETa values in rocky desertification areas.

Classification	PAW	N
NDVI≤0.1	7.22–8.40	5
0.1<NDVI≤0.2	3.82–4.17	2.2
0.2<NDVI≤0.3	2.49–2.63	2.5
0.3<NDVI≤0.4	1.82–1.93	2.2
0.4<NDVI≤0.5	1.44–1.52	2.1
0.5<NDVI≤0.6	1.14–1.21	2.5
0.6<NDVI≤0.7	1.03–1.09	1.8
0.7<NDVI≤1	0.92–0.94	2

Furthermore, we explored the impacts of the NDVI changes on the vegetation ecological water consumption changes. For each severity category of rocky desertification areas, we changed the NDVI amount and kept the precipitation the same and then further modeled the changes in ETa in response to the NDVI changes. For example, if the NDVI of the study area increased to 0.025, 0.05, 0.075 or 0.1 in the future, the ETa changes in the different rocky desertification areas were calculated using the Bagrov model prediction.

**Fig 7:**
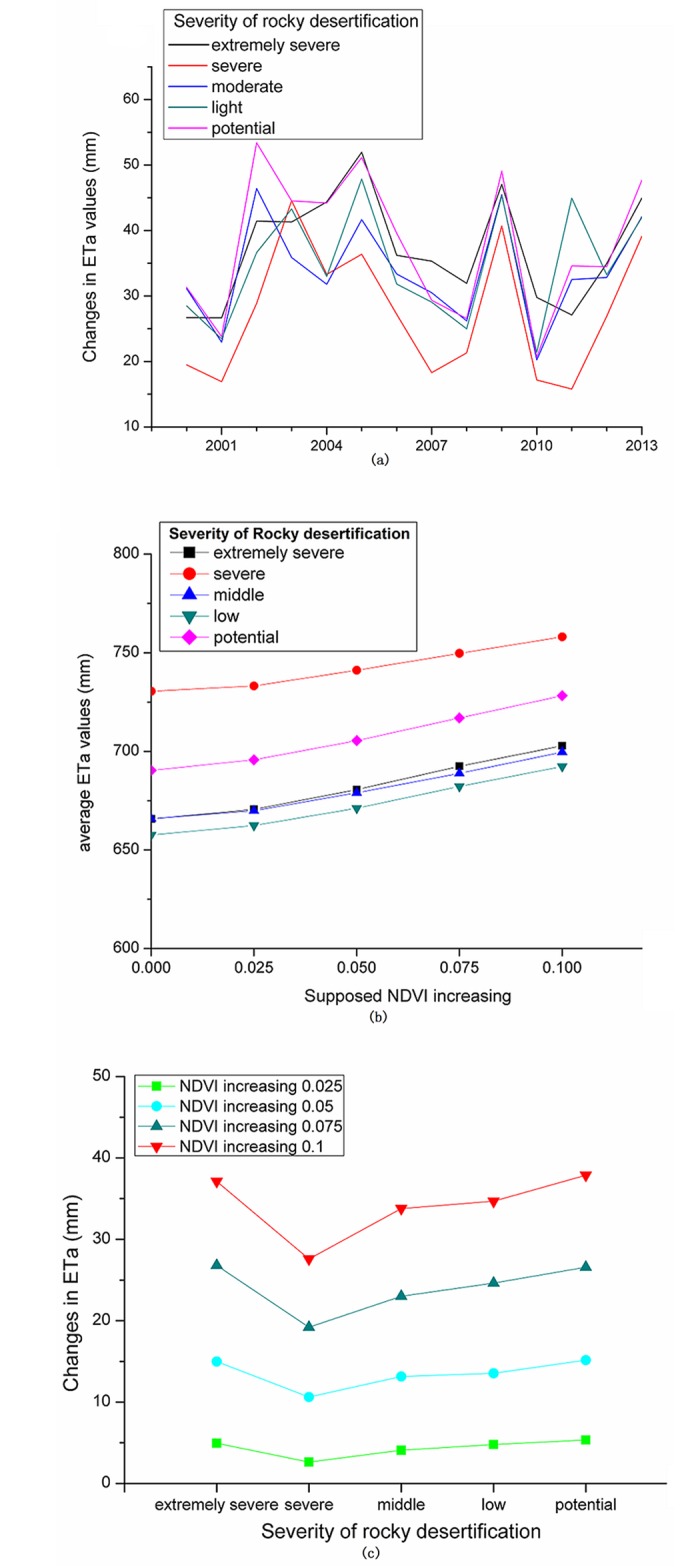
ETa values responses to increasing NDVI for different severity categories of rocky desertification. (**a**) changes in ETa values with NDVI increasing of 0.1 from 2000–2013; (**b**) average annual ETa values with NDVI increasing of 0.025, 0.05, 0.075, and 0.1; and (**c**) changes in average annual ETa values with NDVI increasing of 0.025, 0.05, 0.075, and 0.1.

The results indicated that the ETa response to NDVI changes were obviously different under the different precipitation and different rocky desertification conditions. For example, with the NDVI increasing of 0.1, Fig 7a showed that the ETa would increase of approximately 20–60 mm. In the flooding conditions, as in 2008, the ETa would only increase 20–30 mm in these areas. However, when the extreme drought occurred, the vegetation ecological water consumption response to the NDVI changes were more sensitive, and the ETa could increase 40–60 mm in all of the rocky desertification areas, with the NDVI increasing of 0.1.

The annual ETa values response to NDVI changes were also different due to the different degrees of rocky desertification (Fig 7b). The responses of ETa to NDVI changes were more sensitive in the potential and extremely severe rocky desertification areas. For the severe rocky desertification areas, which had higher precipitation levels (average of 1393 mm), the ETa only increased by an average of 2.64 mm, 10.62 mm,19.19 mm, and 27.58 mm with the NDVI increasing by 0.025, 0.05, 0.075, and 0.1 (Fig 7c). However, for the potential and extremely severe rocky desertification areas, where the precipitation was lower, the water consumption could increase more. For example, with the NDVI increasing of 0.025, 0.05, 0.075, and 0.1, the corresponding ETa changes could increase by averages of 4.94 mm, 14.99 mm, 26.80, and 37.13 mm, respectively, in the extremely severe rocky desertification areas and by averages of 5.34 mm, 15.15 mm, 26.57 mm, and 37.89 mm, respectively, in the potential rocky desertification areas. It is more important to consider the water limitation of the vegetation growth and restoration in the extremely severe rocky desertification areas, where the vegetation was seriously degraded and the precipitation was lower but the vegetation water consumption was more sensitive to NDVI changes.

## Discussion

A comprehensive rocky desertification treatment project was conducted by the government for more than 10 years in South China, and tens of millions mu (1 mu≈666.7 m^2^) of trees were planted in the rocky desertification areas. The project played key roles in vegetation restoration, water and soil conservation and in improving the ecological environment in the rocky desertification areas. However, the increase in regional artificial vegetation greatly disturbed the balance between the water supply and demand in the regional ecosystem. This paper explored the remarkable effects of large-scale vegetation restoration on the increase of vegetation ecological water consumption. In a certain sense, it proved that the continuation of excessive vegetation restoration may cause a water supply deficiency for the regional vegetation ecosystem, consequently limiting the vegetation growth. The government cannot ignore the vegetation ecological water consumption rise in the rocky desertification areas. This study provided a necessary suggestion that the government must pay close attention to the adverse effects on water stresses in the regional vegetation ecosystem due to the vegetation ecological water consumption rise. Indeed, in some rocky desertification areas, excessive vegetation restoration and unsuitable tree species have caused unsuccessful vegetation restorations.

Some researchers have studied the ETa changes’ responses to vegetation coverage changes in the non-karst regions [51, 52]. However, the key finding in this paper was different from other studies, as this paper focused on the karst areas with distinctive topographic features and a very fragile ecosystem. In this region, how the ETa responds to vegetation changes was not clear. We also distinguished the ETa changes responses to vegetation coverage changes among the different severity categories of rocky desertification areas, under the background of a large-scale vegetation restoration by the government. According to our study, the ecology water consumption response to vegetation coverage changes was lower sensitive in severe rocky desertification areas. The main reason we considered was the higher precipitation. However, many factors influencing the ecology water consumption changes in karst area, such as precipitation, temperature, vegetation, exposed bedrock and soil types. We think that the heterogeneity of the microhabitat, rock outcrops redistribute water might greatly influence the soil water distribution and consequently impact on the vegetation water consumption variation in the different rocky desertification areas [53, 54]. Clarifying the reason for the different response of the ETa to vegetation variation in different rocky desertification areas needs more controlled experiments.

In the extremely severe rocky desertification areas, the ecology water consumption response to vegetation coverage changes was highly sensitive. The vegetation restoration project needs to carefully consider the water limitation to the vegetation growth. It is necessary to choose the low water consumption plant species and improve water use efficiency in this area. To carry out the vegetation restoration more rationally, we need to further estimate the revegetation, potentiality under the water limiting conditions in the different rocky desertification areas.

Also, our study has the limitations. Though the Bagrov model can easily simulate the ETa for the region and has the advantage of less data needs, but it is an empirical model. The model combined with MODIS NDVI cannot simulate the delicate processes for the energy balance and difficult for finding the mechanism of the ETa response to the vegetation coverage changes. Clarifying the relationship between MODIS NDVI and the ETa needs more controlled experiments in the karst area. We will further do these studies in our future researches.

## Conclusions

Water for vegetation growth is very important for the ecological sustainable development in the rocky desertification karst areas. However, the vegetation restoration can lead to a rise in ecological vegetation water consumption. The main conclusions of our study were as follows:

The evapotranspiration efficiency (ETa/ETp) and potential humidity (P/ETp) values were greatly influenced by the precipitation and NDVI changes and were more sensitive in the lower precipitation conditions. The revised Bagrov model used in our study can correctly reflect the variation of the vegetation ecological water consumption (ETa) in the rocky desertification areas. The vegetation ecological water consumption changes were more sensitive when the precipitation values were lower. When a drought occurred, the ETa could increase of 40–60 mm in the rocky desertification areas, with the NDVI increasing of 0.1. The responses of the ETa to vegetation coverage changes were lower in severe rocky desertification areas, but more sensitive in the potential and extremely severe rocky desertification areas.
